# Therapeutic plasma exchange in critically ill patients in low-income and lower-middle-income countries: medical need and feasibility

**DOI:** 10.7189/jogh.15.04214

**Published:** 2025-07-25

**Authors:** Rosita Bihariesingh-Sanchit, Rakesh Bansie, Angélique Bastienne van ‘t Wout, Rocade Ma, Dimitri Adriaan Diavatopoulos, Marien Isaäk de Jonge, Arno Pieter Nierich

**Affiliations:** 1 Department of Anesthesiology, Academic Hospital Paramaribo, Paramaribo, Suriname; 2 Department of Intensive Care, Academic Hospital Paramaribo, Paramaribo, Suriname; 3 Department of Internal Medicine, Academic Hospital Paramaribo, Paramaribo, Suriname; 4 AlphaBiomics Limited, Newcastle upon Tyne, UK; 5 Van ‘t Wout Pharma Consulting, Amsterdam, the Netherlands; 6 Department of Laboratory Medicine, Laboratory of Medical Immunology, Radboud University Medical Center, Nijmegen, the Netherlands; 7 Radboud Community for Infectious Diseases, Radboud University Medical Center, Nijmegen, the Netherlands; 8 HemoClear BV, Zwolle, the Netherlands

## Abstract

**Background:**

Therapeutic plasma exchange (TPE) is a blood purification technique designed for the removal of large molecules such as pathogenic antibodies and lipoproteins. The procedure involves removing plasma from the patient in exchange for replacement fluid, and it can be performed either by membrane separation or centrifugation. These conventional techniques are expensive and require the training of skilled personnel. This severely limits their use in low-income countries (LICs) and lower-middle-income countries (LMICs), leading to morbidity and mortality for patients in LICs and LMICs suffering from the diseases where TPE is indicated.

**Methods:**

A novel gravity-driven blood separation method might provide the needed access to TPE for LICs and LMICs. We reviewed the medical need, the practical aspects, as well as the possible complications of applying this novel technology in LICs and LMICs. Furthermore, we describe a feasibility study of implementing TPE in Suriname for various diseases and conditions.

**Results:**

Where data was available (n /N = 10/11), supportive care combined with TPE using the new device resulted in improved values for the disease-specific markers evaluated in these patients. In addition, eight patients showed complete clinical recovery, and one patient showed partial clinical recovery upon TPE within 0.5–6 months of follow-up. Importantly, none of the patients experienced any serious side effects.

**Conclusions:**

This experience in the resource-limited setting in Suriname illustrates that its application is feasible in LICs and LMICs settings, at least for these five diseases with first-line indications for TPE and a significant number of patients.

Plasmapheresis is the removal, treatment, and return or exchange of blood plasma or components thereof from and to the blood circulation [1]. Therapeutic plasma exchange (TPE), where plasma is removed from the patient in exchange for replacement fluid, can decrease the levels of unwanted molecules [2,3]. This procedure has great value in critically ill patients, specifically in severe cases of systemic inflammatory responses, organ dysfunction, and refractory conditions [4]. However, TPE remains largely inaccessible in low-income countries (LICs) and lower-middle-income countries (LMICs) due to high costs, inadequate infrastructure, and the need for specialised equipment and trained personnel [5].

We aim to explore the medical needs for TPE in critically ill patients in LICs and LMICs, discuss the currently available options, and present findings from an implementation feasibility study in Suriname applying a novel gravity-driven TPE method in critically ill patients with no access to conventional treatments. By sharing these results, we aim to contribute to a more affordable and scalable approach to therapeutic apheresis in resource-limited settings.

The American Society for Apheresis (ASFA) reviews, updates, and categorises indications for the evidence-based use of therapeutic apheresis in human disease. The most recent (ninth) version of the guidelines comprises 91 conditions with 166 graded and categorised indications based on the quality of the supporting evidence [4], and TPE is accepted as first-line therapy with evidence grades 1A–C for 15 of these 91 conditions (**Table 1**). However, most of the evidence used in the ASFA guidelines is from studies in high-income countries, and the recommendations do not distinguish between countries, even though the setting may heavily influence implementation, accessibility and efficacy [5]. This highlights the pressing need to evaluate practical and affordable approaches to delivering TPE in resource-constrained environments, particularly for critically ill patients where timely intervention can be lifesaving.

**Table 1:** Disorders for which therapeutic plasma exchange is accepted as first-line therapy with evidence grade 1A–C*

Disease/condition by modality†	Indication‡	Evidence grade§	Patient case¶
Acute inflammatory demyelinating polyradiculopathy (GBS)			
*TPE*	Primary treatment	1A	Yes
Acute liver failure			
*TPE*	All	1A	Yes
Anti-glomerular basement membrane disease (Goodpasture syndrome)			
*TPE*	DAH	1C	
*TPE*	Dialysis independence	1B	
Chronic acquired demyelinating polyneuropathies			
*TPE*	IgG/IgA/IgM related (only)	1B	
Chronic inflammatory demyelinating polyradiculoneuropathy			
*TPE/IA*	All	1B	
Familial hypercholesterolemia			
*TPE/LA*	Homozygotes	1A	Yes
Focal segmental glomerulosclerosis			
*TPE/IA*	Recurrent in kidney transplant	1B	
Hyperviscosity in hypergammaglobulinemia			
*TPE*	Symptomatic	1B	
*TPE*	Prophylaxis for rituximab	1C	
Myasthenia gravis			
*TPE/DFP/IA*	Acute, short-term treatment	1B	Yes
N-methyl-D-aspartate receptor antibody encephalitis			
*TPE/IA*	All	1C	
Thrombotic microangiopathy, thrombotic thrombocytopenic purpura			
*TPE*	All	1A	
Transplantation, liver			
*TPE*	Desensitisation, ABOi living donor	1C	
Transplantation, kidney, ABO compatible			
*TPE/IA*	Antibody-mediated rejection	1B	
*TPE/IA*	Desensitisation/prophylaxis, living donor	1B	
Transplantation, kidney, ABO incompatible			
*TPE/IA*	Desensitisation, living donor	1B	
Wilson disease, fulminant			
*TPE*	All	1C	Yes

DAH – diffuse alveolar haemorrhage, DFP – double filtration plasmapheresis, GBS – Guillain-Barré syndrome, IA – immunoadsorption, LA – lipoprotein apheresis, TPE – therapeutic plasma exchange

*Adapted from Connelly-Smith et al. 2023 [4].

†Disease refers to a specific disease (*e.g.* myasthenia gravis), and condition refers to a specific medical condition (*e.g.* transplantation, liver).

‡Indication refers to the use of TPE in specific situations encountered in the disease/condition (*e.g.* for myasthenia gravis (disease), acute, short-term treatment (indication)).

§Based on the GRADE system [6]: 1A – Strong recommendation, high-quality evidence, 1B – Strong recommendation, moderate quality evidence, 1C – Strong recommendation, low-quality or very low-quality evidence.

¶Patient(s) treated for specified disease indication using therapeutic apheresis modality at Academic Hospital Paramaribo, Paramaribo, Suriname (Table S1 in the **Online Supplementary Document**).

Currently, two TPE technologies are available in high-income countries: centrifugation and membrane filtration [7–9]. Centrifugal-based techniques utilise centrifugal force to separate whole blood into components of varying densities, allowing low-density plasma to be collected for removal or donation. Membrane-based techniques use membranes manufactured with specific pore sizes, allowing the passage of specific blood components while retaining cells and larger components. Both systems are comparable, adequate, and safe. Although both systems replace fluid at an equal or greater volume than what was removed, membrane filtration TPE extracts a smaller fraction of plasma (30%) per unit of time than centrifugal systems (80%), requiring longer treatment times and higher blood flow rates [10]. Although the two TPE technologies are established and upscaled, challenges related to the techniques’ selectivity and long-term impact on blood cells and their constituents remain. The centrifugal forces or high flow rates through a membrane during extraction may stress blood cells, leading to cell damage, haemolysis, and platelet activation, and may harm the donor plasma [8].

The availability and accessibility of TPE using either of the two technologies in LICs and LMICs can be limited by various factors, including the high cost of capital equipment and consumables, lack of trained personnel, and inadequate healthcare infrastructure. The initial capital costs for conventional TPE equipment range between USD 50 000 and 150 000 per unit [11]. Furthermore, a standard treatment of six TPE procedures has recently been estimated to cost between USD 14 628 and 17 856, excluding additional expenses such as intravenous immunoglobulin (IVIG), which can cost USD 915 per infusion [12]. While conventional TPE is available in countries such as India [13], Nepal [14], Malaysia [15], and Nigeria [16], it is typically limited to a small number of tertiary centres and not accessible to large sections of the population, particularly in rural and underserved areas. To address the high costs associated with TPE, some LMICs have adopted cost-effective technologies and locally manufactured equipment [17–20]. For example, in India, two groups have reported the successful reuse of plasmapheresis filters to reduce costs, resulting in cost savings per session of around 45% [21] and 57% [22]. While beneficial in specific contexts, filter reuse must be approached with caution as proper sterilisation and validation protocols are essential to ensure patient safety. Separately, in India and Bangladesh, an alternative approach known as small-volume plasma exchange is used. This is a simplified technique that enables the local production of elements, based on the same principle as conventional plasma exchange, but at significantly lower costs (approximately USD 500 per patient). It involves four to six daily sessions of whole blood sedimentation by gravity, followed by removal of supernatant plasma after retransfusion of the sedimented blood cells with a target of removing an overall volume of at least 8 L of plasma over a total of eight days [17,18,23,24]. However, small-volume plasma exchange is a time-consuming and labour-intensive procedure. Moreover, because of the frequent sessions (*i.e.* on average 30 per patient), there are increased risks of sepsis, insertion site bleeding, as well as replacement fluid hypersensitivity and transient hypotension.

Several LMICs have initiated training programmes and collaborations with international organisations to build local capacity and expertise in TPE [25,26]. However, the implementation of TPE also requires specific infrastructure and logistics, including the availability of replacement fluids (*e.g.* albumin solution or fresh-frozen plasma) and appropriate storage facilities. In LICs and LMICs, there are often shortages of these replacement fluids, necessitating the exploration of alternative solutions, such as the use of locally sourced plasma or volume expanders [25]. Given these persistent barriers to conventional TPE implementation in resource-limited settings, we aimed to explore the feasibility of an alternative gravity-driven TPE method suitable for use in LICs and LMICs.

## METHODS

### Gravity-driven membrane-based TPE in a resource-limited setting

The above barriers underscore the necessity for more cost-effective and safe alternatives to conventional plasmapheresis techniques, which demand lower operational expertise and can be deployed in LICs and LMICs. To this end, we have successfully implemented the use of an alternative membrane-based bedside plasmapheresis device (HemoClear) to obtain COVID-19 convalescent plasma and treat intensive care patients admitted in Suriname [27,28].

**Figure 1:**
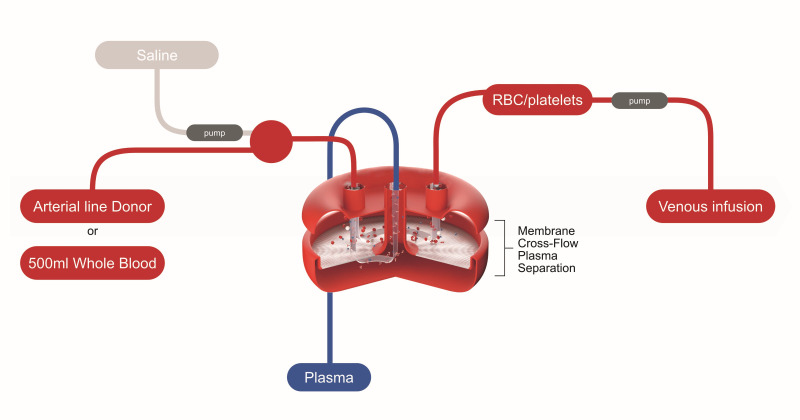
Schematic diagram of the HemoClear blood separation process.

HemoClear is a gravity-driven, membrane-based, crossflow microfiltration blood product separation device (**Figure 1**; Video S1 in the **Online Supplementary Document**), initially piloted in 2018 for washing shed blood in cardiac surgery [29]. Its success in bedside apheresis, cost-effectiveness, and practicality led to its implementation for the collection of other blood products, including plasma for convalescent plasma treatment. The HemoClear device separates whole blood into concentrated blood cells and plasma by passing blood through a multilayered crossflow membrane module, with a relatively large membrane pore size of 2.3 μm. The closed-system filtration process prevents potential contamination [30]. The device is portable, disposable, and contains no electrical components. The microfiltration process occurs solely through the force of gravity, either generated by the donor's mean arterial pressure or by positioning the donor blood one meter above the filter on an infusion rod [30]. The procedure for filtration of one litre of whole blood takes 30 minutes and does not require a significant investment, electricity, extensive training, or costly consumables [31]. A combination of one hour of online instruction and one day of on-site training is sufficient to train local staff to use the HemoClear device effectively. Importantly, at USD 400 per treatment, the cost of TPE using the HemoClear method is at least three times cheaper than the conventional methods that range between USD 1200 and 3000 per treatment [32,33] with extremes as high as USD 10 000 [34]. Combined, these features render the HemoClear method ideal for use in resource-constrained environments, such as those in poor, rural, or casualty settings where an uninterrupted power supply is not guaranteed.

### Implementation feasibility study of gravity-driven TPE in critically ill patients in Paramaribo, Suriname

**Figure 2:**
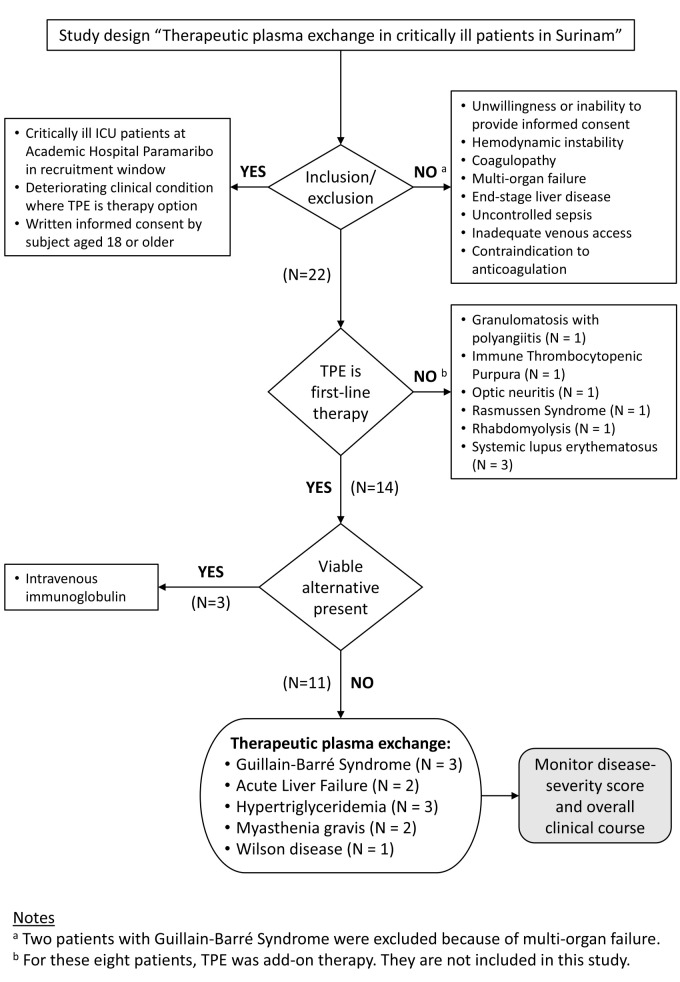
Flowchart with study enrolment and experimental design.

Using this gravity-driven filtration approach, we are conducting an implementation feasibility study for the use of TPE in the 15 first-line TPE indications identified in the ASFA guidelines with evidence grade 1A–C (**Table 1**). We followed the Strengthening the Reporting of Observational Studies in Epidemiology guidelines in our study [35]. The study flowchart illustrates the study enrolment and experimental design (**Figure 2**). We collected a prospective case series in which treatment decisions were based on clinical necessity in the absence of conventional options. Critically ill patients admitted to the Academic Hospital Paramaribo between June 2022 and June 2024 were considered for inclusion if they had a deteriorating clinical condition for which TPE was indicated (**Table 1**), and no viable treatment alternatives were available. Other inclusion criteria included written informed consent from the patient and a minimum age of 18 years. Exclusion criteria were unwillingness or inability to provide informed consent, hemodynamic instability, coagulopathy, multi-organ failure, end-stage liver disease, uncontrolled sepsis, inadequate venous access, and contraindication to anticoagulation. In this period, two intensive care unit (ICU) patients with Guillain-Barré syndrome (GBS) had very advanced stages of the disease (GBS disability score = 5), and TPE was given only for compassionate use and were not included in the study. Eight ICU patients had conditions where TPE was not the first-line therapy and were not included in the study. Three ICU patients received IVIG as a viable treatment alternative and were not included in the study. No ICU patients were excluded from the series after enrolment.

The team had prior experience with the HemoClear method for plasma collection and could safely adapt it for TPE. All treating physicians and nurses in the study were formally trained in TPE using the HemoClear device prior to study initiation, following established critical care protocols. Training included didactic sessions, hands-on workshops, and competency evaluations to ensure procedural proficiency. Clinicians adhered to standard operating procedures for device setup, adverse event monitoring, and troubleshooting.

We recorded demographic, clinical, and laboratory data in real time; the data were not extracted retrospectively from patient records. At least one other qualified individual independently reviewed the data entry to ensure accuracy. The patient’s neurologist or internal medicine physician determined the initial disease score, and the intensivist or ICU physician subsequently re-evaluated it. Trained clinicians performed all scoring in accordance with established clinical competency guidelines. We defined complete clinical recovery as the patient’s ability to resume all pre-admission activities, including work. We analysed all blood samples in a single laboratory setting using standardised testing protocols to ensure consistency and reliability.

We defined adverse events as any undesirable experience occurring to a patient during the study. We systematically documented these events following the protocols of the Academic Hospital Paramaribo. Serious adverse events – defined as prolonged hospitalisation, persistent disability, or death – were closely monitored. The lead investigator (RBA) was responsible for reporting these events to the accredited ethics committee and competent authorities. Patient records related to adverse events are maintained for a minimum of 15 years post-trial.

To date, we have treated patients for five of the 15 indications: acute inflammatory demyelinating polyradiculopathy (*i.e.* GBS), acute liver failure (ALF), hypertriglyceridemia, myasthenia gravis (MG), and Wilson disease (WD) (Table S1 in the **Online Supplementary Document**). This demonstrates the initial feasibility of using TPE in a resource-limited setting for indications with significant incidence and prevalence in LICs and LMICs (**Table 2**).

**Table 2:** Incidence, prevalence and projected number of patients by World Bank country classification

			Projected number of patients per year*				
	**Incidence(per million individuals per year)**	**Prevalence(per million individuals per year)**	**HICs (n = 83)**	**UMICs (n = 54)**	**LMICs (n = 54)**	**LICs (n = 26)**	**Total (n = 218)**
**GBS**	10–20†	0.8–6.43‡	18 857	38 063	52 736	11 675	121 331
**ALF**	<10†	NA	6286	12 688	17 579	3892	40 444
**FH**	NA	1–6†	4400	8881	12 305	2724	28 311
**MG**	7–23†	124§	18 857	38 063	52 736	11 675	121 331
**WD**¶	1–2†	6–7║	1823	3679	5098	1129	11 729
**TOTAL**			50 222	101 375	140 454	31 095	323 164

ALF – acute liver failure, FH – familial hypercholesterolemia, GBS – Guillain-Barré syndrome, HICs – high-income countries, LICs – low-income countries, LMICs – lower-middle-income countries, MG – myasthenia gravis, NA – not applicable, UMICs – upper-middle-income countries, WD – Wilson disease, x̄ – mean

*Calculated by multiplying each country group's population [36] by the global incidence of each disease (as tabulated in the second column). Countries are grouped according to the World Bank Classification [37].

†From Connelly-Smith et al. 2023 [4]. There is no incidence data available for FH ; therefore, prevalence is used for the calculation of projected patient numbers.

‡From Global Health Data Exchange [38].

§From Salari et al. 2021 [39].

¶Fulminant Wilson disease cases only (around 5% of all patients).

║From Gao et al. 2019 [40].

## RESULTS

### Case studies in which TPE was used in critically ill patients in a resource-limited setting

We collected a prospective case series of patients admitted between June 2022 and June 2024 to the Academic Hospital Paramaribo in Paramaribo, Suriname, requiring TPE for non-renal indications. We collected demographic data, clinical data, details of treatment for primary diseases, plasma exchange procedure details, complications of procedure, outcome, and follow-up details.

Eleven patients underwent TPE during this two-year period with a male to female ratio of 4:7 and a mean (x̄) age of 42 years (range = 26–68 years) (Table S1 in the **Online Supplementary Document**). We categorised and graded the indications for plasma as per the recent ASFA 2023 guidelines [4] on the use of TPE in clinical practice (**Table 1**). We conducted necessary laboratory and clinical investigations to establish the respective diagnoses.

For the TPE treatment, patients were given both an arterial line and a peripheral infusion line. The arterial line – combined with lines feeding the washing solution (0.9% NaCl) and heparin (10 000 units over two hours) – was connected to the first port of the HemoClear filter via a perfusion pump set at 200–250 mL per hour. We collected plasma from the second filter port and the concentrated cell fraction (primarily red blood cells) from the third filter port and then re-infused via the peripheral infusion line. Plasma volume exchanged ranged from 400–1800 mL (x̄ = 1113 mL) in 1–9 sessions (x̄ = 5). Patient’s plasma was replaced with fresh frozen plasma and/or albumin solution (200 g/L) (only one type per session). Along with plasma exchange, patients received supportive treatment as indicated by their specific condition. To determine the effect of TPE treatment, patients were monitored using disease-specific markers: GBS disability score [41] for patients with GBS, blood ammonia levels for patients with ALF, blood triglyceride levels for patients with hypertriglyceridemia, MG activities of daily living scale [42] for patients with MG, and Glasgow coma scale score [43] for patients with WD.

### GBS

Acute inflammatory demyelinating polyradiculopathy, commonly known as GBS, is an acute polyneuropathy that can lead to the rapid onset of muscle weakness and paralysis. It is the major cause of acute non-traumatic paralysis in healthy individuals and is thought to be caused by autoimmune reactions induced by infection with viral (*e.g.* influenza virus, cytomegalovirus, Epstein-Barr virus, COVID-19 virus) or bacterial pathogens (*e.g. Campylobacter jejuni*, *Mycoplasma pneumoniae*) [44]. The effectiveness of TPE in reducing the duration and severity of symptoms is due to its ability to remove circulating auto-reactive antibodies that attack the peripheral nervous system. The worldwide prevalence of GBS ranges between 0.8 and 6.4 cases per million, with multiple LMICs in Middle and South America, and Southern Africa having prevalences above two cases per million [38]. The incidence of GBS is estimated at 10–20 per million per year [4] for a projected total of >64 000 GBS patients per year in LICs and LMICs (**Table 2**).

**Figure 3:**
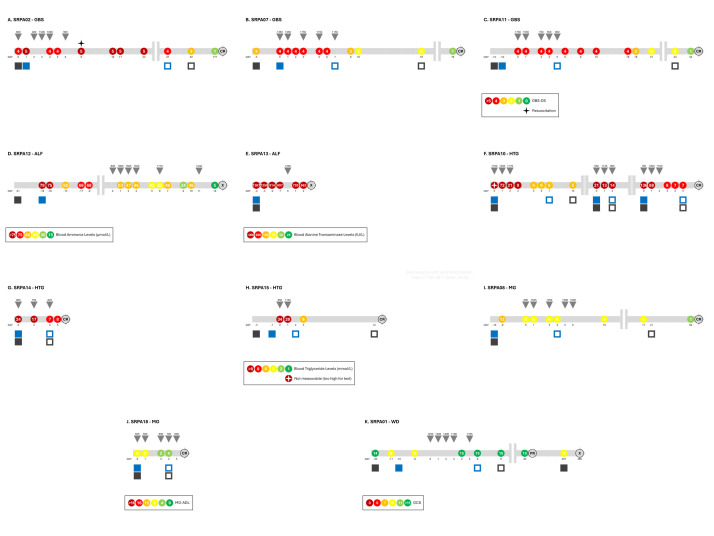
Visual timeline for each patient included the implementation feasibility study. ALF – acute liver failure, CR – complete recovery, GBS – Guillain-Barré Syndrome, HTG – hypertriglyceridemia, MG – myasthenia gravis, PR – partial recovery, WD – Wilson disease, X – deceased. **Panels A−C.** Three GBS patients with disease severity reported on GBD-DS scale. **Panels D−E. **Two ALF patients with disease severity reported using blood ammonia or alanine transaminase levels. **Panels F−H. **Three HTG patients with disease severity reported using blood triglyceride levels. **Panels I−J.** Two MG patients with disease severity reported on MG-ADL scale. **Panel K. **One WD patient with disease severity reported on GCS scale. Each timeline also shows hospital (filled black square) and ICU (filled blue square) admission and hospital (open black square) and ICU (open blue square) discharge dates, TPE treatment sessions (gray triangle), and overall treatment outcome (complete recovery, partial recovery or deceased). Number above the gray triangle indicates plasma volume (mL) exchanged. Time is depicted in days relative to the start of TPE.

Three patients with GBS, SPRA02, SPRA07, and SPRA11 (**Figure 3**, Panels A–C; Table S1 in the **Online Supplementary Document**), underwent five sessions each of TPE upon admission to intensive care for suspected GBS. All three showed clear neurological improvement with TPE treatment (reflected in decreased GBS disability scores), and all patients have made a complete recovery within six months of follow-up.

### ALF

ALF is the rapid onset of severe complications following the first signs of liver disease, indicating that the liver is severely damaged. The development of ALF can occur in a normal liver or in the setting of chronic liver disease. The mortality rate in ALF is estimated at 50–90% due to acute metabolic disturbances, hepatic encephalopathy, and severe coagulopathy resulting in multiple organ failure. In ALF, TPE can remove albumin-bound or unbound toxic components (*e.g.* drugs and mushroom-derived toxins) as well as inflammatory factors responsible for organ failure, hepatic encephalopathy, and decreased systemic vascular resistance and cerebral blood flow. As an adjunct or standalone therapy, TPE has been used for bridging patients to recovery or liver transplantation. The incidence of ALF is around one case per 100 000 [4], for a projected total of >21 000 patients per year in LICs and LMICs (**Table 2**).

Two patients with ALF, SPRA12 and SPRA13 (**Figure 3**, Panels D–E; Table S1 in the **Online Supplementary Document**), underwent TPE for severe encephalopathy as observed by electroencephalography. Ammonia levels were measured in one patient (SPRA12), showing improvement with TPE treatment. However, in both cases, the patient’s condition continued to deteriorate, and both patients succumbed to multi-organ failure within 6–45 days after hospital admission (2–14 days after initiation of TPE).

### Familial hypercholesterolemia

Familial hypercholesterolemia (FH) is a common genetic cause of premature atherosclerotic cardiovascular disease, caused by mutations in genes involved in the low-density lipoprotein receptor pathway. Patients with homozygous or heterozygous FH represent high-risk phenotypes, at risk for developing coronary heart disease before 18 or 55 years of age, respectively. However, once diagnosed, lipid-lowering treatment successfully prevents cardiovascular events and reduces mortality. In cases of severe hypercholesterolemia resistant to conventional therapy (substantial in homozygous FH), plasmapheresis and specifically lipoprotein apheresis can be used as an adjunctive therapy to reduce lipid levels rapidly. The worldwide prevalence of homozygous FH is estimated at 1–6 cases per million [4], resulting in a projected >15 000 cases in LICs and LMICs (**Table 2**).

We did not identify patients with FH for our prospective case series. However, three patients, SPRA10, SPRA14, and SPRA15 (**Figure 3**, Panels F–H; Table S1 in the **Online Supplementary Document**), with pancreatitis and markedly elevated triglyceride levels, underwent 2–9 TPE sessions each. One patient (SRPA10) underwent three TPE sessions on three separate occasions, between September 2022 and December 2024 (**Figure 3****,** Panel F). For the summary statistics, only the results of the first set of TPE sessions are included. In these patients we could assess the effect of the TPE treatment on blood triglyceride (**Figure 3**, Panels F–H; **Table 3**) as well as cholesterol levels (SPRA10 = 22.23 > 4.4 mmol/L; SPRA14 = 22.45 > 12.77 mmol/L; SPRA15 = 13.82 > 7.79 mmol/L), showing sharp decreases with treatment in all three patients who also improved clinically and were discharged from the hospital within 14 days after initiation of TPE.

**Table 3:** Recovery trend by condition*

	Patients, n	Disease marker	Marker improvement, %	ICU stay, in days	Hospital stay, in days	Recovery type	Time to recovery, in months
**GBS**	3	GBS-DS	75 (75–75)	7 (5–37)	34 (18–52)	Complete	2.5 (1.8–5.9)
**ALF**	2	Ammonia (μmol/L)†	93	NA	NA	Deceased	NA
**HTG**	3	Triglycerides (mmol/L)	82 (66–91)	4 (2–7)	12 (4–14)	Complete	0.40 (0.17–0.47)
**MG**	2	MG-ADL	67 (50–83)	4 (4–4)	13 (4–21)	Complete	1.2 (0.17–2.1)
**WD**	1	GCS	67	6	9	Partial	1.0

ALF – acute liver failure, GBS – Guillain-Barré syndrome, GBS-DS – Guillain-Barré syndrome disability score [33], GCS – Glasgow coma scale [41], HTG – hypertriglyceridemia, ICU – intensive care unit, MG – myasthenia gravis, MG-ADL – myasthenia gravis activities of daily living scale [34], NA – not applicable, WD – Wilson disease

*Values are presented as medians (ranges) where applicable.

†Blood ammonia levels are available for one ALF patient only.

### MG

Autoimmune disease myasthenia gravis (MG) is characterized by muscle weakness resulting from autoantibodies targeting the acetylcholine receptors at the neuromuscular junction. These antibodies induce fluctuating weakness of skeletal muscles, which typically worsens later in the day, after repetitive muscle use, or after exercise. Regional or focal, MG usually involves the eye muscles, which causes diplopia and ptosis. In cases that are severe or refractory, TPE is used to decrease the levels of these autoantibodies rapidly. The worldwide prevalence of MG is 124 cases per million [39]. Incidence of MG is estimated at 7–23 cases per million per year [4] for a projected total of >64 000 MG patients per year in LICs and LMICs (**Table 2**).

Two patients with MG, SPRA08 and SPRA18 (**Figure 3**, Panels I and J; Table S1 in the **Online Supplementary Document**), underwent five sessions each of TPE upon admission to intensive care for MG disease exacerbations. Both showed clear neurological improvement with TPE treatment (as reflected in sharply decreased MG disease scores), and both patients have made a complete recovery within 2.5 months of follow-up.

### WD

Hepatolenticular degeneration, known as WD, is an autosomal recessive disorder caused by abnormal copper accumulation in the body, particularly involving the brain, liver, and cornea. The disease usually presents between the ages of 5 and 35. Children typically present with asymptomatic liver deposits of copper, teenagers with liver disease, and adults with neurological symptoms. Liver transplantation is potentially curative, reversing most of the clinical and biochemical pathological manifestations of the disease within months, and is the mainstay of therapy for patients with ALF due to WD. As a bridge to liver transplantation, TPE is used to remove significant amounts of copper from the circulation rapidly. The worldwide prevalence of WD is 127–139 cases per million [40]. Incidence of WD is estimated at 25–33 cases per million per year [4], of whom approximately 5% develop ALF [45], resulting in a projected total of >6200 patients per year who are indicated for TPE in LICs and LMICs (**Table 2**).

One patient with WD, SPRA01 (**Figure 3**, Panel K; Table S1 in the **Online Supplementary Document**), underwent five sessions of TPE after admission to the intensive care unit for progression of WD. Patient symptoms of coma and convulsions improved initially (reflected in increased Glasgow coma scale scores), and she was discharged from the hospital. However, she succumbed to her illness within ten months after her initial admission.

## DISCUSSION

Where data were available (for 10 of the 11 patients), supportive care combined with TPE using the HemoClear device resulted in improved values for the disease-specific markers evaluated in these patients (**Table 3**). In addition, eight patients showed complete clinical recovery, and one patient showed partial clinical recovery upon TPE within 0.5 to six months of follow-up. The two patients suffering from ALF continued to deteriorate despite treatment and finally succumbed to multi-organ failure. However, one patient recovered consciousness after TPE, allowing contact with the family members, suggesting the potential value of compassionate use of TPE under certain circumstances. Importantly, none of the patients experienced any serious side effects (such as insertion site bleeding, fresh frozen plasma hypersensitivity, or hypotension) from TPE treatment using the HemoClear device in this setting. While the number of patients per indication in this implementation feasibility study (1–3 per indication) is too small for formal conclusions, the outcomes are generally consistent with those reported for these five indications with conventional TPE (**Table 3**) [4].

The above indicates that TPE using the HemoClear device is feasible in LICs and LMICs settings, at least for diseases with first-line indications for TPE. Extrapolating to other LICs and LMICs combined, the five diseases have a projected patient number of over 170 000 (**Table 2**), showing the medical need that could already be addressed by implementing this method of TPE.

The experience with TPE using the HemoClear device in the resource-limited setting in Suriname illustrates that its application is feasible in an LIC and LMIC setting, at least for these five diseases with first-line indications for TPE and a significant number of patients. However, outcomes varied by indication (**Table 3**). The fastest responses were observed in three pancreatitis patients, all of whom showed marked improvement based on biomarkers within days and were discharged from the hospital within 14 days. Notably, one patient (SRPA10) demonstrated similar rapid biomarker-based improvement on three separate occasions over two years. Moderate but consistent responses were observed in patients with MG and in those patients with GBS, characterised by disease scores of ≤4, with disease score improvements within days to weeks of TPE initiation and complete recovery within six months of follow-up. Of note, two other GBS patients with very severe disease (GBS disease score = 5), hence not eligible for study inclusion, did not respond to TPE and died, highlighting the potential limitations of TPE in very advanced disease stages. Patients with WD and ALF presented greater challenges. The WD patient initially showed clinical improvement following TPE but ultimately succumbed to the disease. Similarly, two patients with ALF demonstrated biomarker-based improvement (reductions in blood ammonia and liver enzyme levels) but showed no clinical benefit and did not survive.

Importantly, none of the patients experienced any serious side effects, providing an advantage over the small-volume plasma exchange method that has also been applied in resource-limited settings [17–19]. We are confident that most of the other first-line indications with evidence grade 1A–C can also benefit from using this gravity-driven filtration approach, thereby addressing an even larger medical need in LICs and LMICs. In addition, second- and third-line TPE indications, such as snake envenomation and exposure to toxins, occur more frequently in LICs and LMICs that lack ready access to the relevant antivenom or antitoxin [46,47]. In those situations, a cost-effective and safe method and thus more easily applicable TPE, could become a more viable option for treatment [48,49].

It is important to note that TPE may impact drug concentrations through direct removal or by inhibiting the removal of metabolising enzymes [10]. Certain drugs, such as acetylsalicylic acid, cefazolin, ceftriaxone, glyburide, heparin, ibuprofen, levothyroxine, valproic acid, verapamil, and warfarin, are particularly amenable to removal by TPE [10]. This is important to consider both for drug dosing and for determining whether TPE can be beneficial in treating intoxications with these drugs. Conversely, some drugs may affect TPE; for example, because angiotensin-converting enzyme inhibitors block the degradation of bradykinins, hypotension can occur if kinins are activated during TPE in patients taking these medications [10].

In summary, TPE offers a valuable treatment option for critically ill patients in LICs and LMICs, addressing a range of severe conditions, including autoimmune diseases, haematological diseases, and other critical illnesses as illustrated by the case series above. Despite the significant challenges in implementing TPE in resource-limited settings, its accessibility and efficacy can be enhanced. Addressing financial constraints, raising awareness, and streamlining logistical processes are key steps toward the successful implementation of TPE in LICs and LMICs. For example, the knowledge of TPE and its applications can be minimal among local professionals, and efforts should also be concentrated on improving awareness and understanding in resource-limited settings [26].

By enabling autologous blood reinfusion and minimising the need for donor blood, especially in resource-constrained environments, the HemoClear method aligns well with the World Health Organization's active support for patient blood management as outlined recently in its guidance [50] and implementation policy brief [51]. Given its potential impact and cost-effectiveness, it could be a promising candidate for a World Health Organization health technology assessment to inform global use.

Several factors inherently limit our study. First, its small size (between one and three patients per indication) limits its generalisability, while the lack of blinding and randomisation may have introduced bias. Additionally, the absence of a direct comparator (*e.g.* IVIG, standard supportive care) limits the ability to estimate clinical and cost-effectiveness. Moreover, the short time frame may not capture scalability, sustainability, and any delayed effects. While we acknowledge the constraints of this limited implementation feasibility study, this situation reflects real-world emergency interventions in resource-limited settings. Reporting our findings may help demonstrate how to offer a practical, cost-effective solution for countries where IVIG and conventional TPE are unavailable or unaffordable. We also hope that this case series can serve as a critical feasibility study for implementation, potentially guiding future larger-scale evaluations. At the Academic Hospital Paramaribo, the HemoClear-based TPE approach has been successfully integrated into standard ICU protocols, ensuring its routine use. It is now covered by insurance providers, markedly improving its financial feasibility. This institutional adoption demonstrates the potential for national scalability. Future efforts should focus on national clinical training programmes to ensure standardised operation across multiple hospitals. Additionally, focus should be placed on government-backed funding mechanisms to subsidise filter procurement and consumable costs, as well as the integration into national transfusion and treatment guidelines to ensure regulatory oversight and long-term sustainability.

## CONCLUSIONS

By focussing on sustainable and context-appropriate solutions, it is possible to enhance the delivery of TPE services and improve patient outcomes in LICs and LMICs. Continued research, advocacy, and investment in healthcare systems are vital to achieving this goal and ensuring equitable access to advanced medical therapies for all patients, regardless of their socioeconomic status or geographic location [5].

## Additional material


Online Supplementary Document
