# Supplementary material for: Therapeutic plasma exchange in critically ill patients in low-income and lower-middle-income countries: medical need and feasibility
Source: J Glob Health. 2025 Jul 25;15:04214. doi: 10.7189/jogh.15.04214 (PMC12290984; doi:10.7189/jogh.15.04214)
Supplement: Online Supplementary Document [file jogh-15-04214-s001.zip › jogh-15-04214-s001.pdf]

Supplement to: Bihariesingh-Sanchit R, Bansie R, van 't Wout AB, Ma R, Diavatopoulos DA, de Jonge MI, Nierich AP. Therapeutic plasma exchange in critically ill patients in low-income and lower-middle-income countries: medical need and feasibility. J Glob Health. 2025;15:04214.

Table S1. Baseline characteristics of patients at admission before therapeutic plasma exchange

Patient ID	Age (years)	Gender	Other comorbidities	Indication for TPE	Disease-specific marker	Creatinine (mmol/L)	ASAT (U/L)	ALAT (U/L)	LDH (U/L)	CRP (mg/L)
SRPA02	31	Female	None	Guillain-Barré Syndrome	GBS-DS = 4	47	N/T	N/T	139	0.6
SPRA07	48	Male	None	Guillain-Barré Syndrome	GBS-DS = 4	74	76	122	195	1.0
SRPA11	68	Female	hypertension, diabetes, hypercholesterolemia, gout, chronic venous insufficiency, obesity	Guillain-Barré Syndrome	GBS-DS = 4	117	11	14	119	11.9
SPRA12	52	Male	hypertension, diabetes, smoking, alcohol abuse	Acute liver failure	Ammonia (NH_3) = 68 µmol/L	210	111	14	279	13.0
SPRA13	42	Female	myocardial infarction, percutaneous coronary intervention, diabetes	Acute liver failure	N/T	25	800	897	722	3.5

SRPA10	34	Female	diabetes, pancreatitis	Hypertriglyceridemia	Triglycerides = 72.1 mmol/L	56	30	19	108	<0.5
SPRA14	33	Female	diabetes, pancreatitis	Hypertriglyceridemia	Triglycerides = 24.0 mmol/L	N/T	N/T	N/T	N/T	N/T
SPRA15	27	Male	alcohol abuse, stomach aches, pancreatitis	Hypertriglyceridemia	Triglycerides = 33.5 mmol/L	N/T	42	43	264	N/T
SRPA08	55	Male	hypertension, diabetes, gout, herniated nucleus pulposus, tuberculosis	Myasthenia gravis	MG-ADL = 6	95	17	14	101	0.5
SRPA18	47	Female	thymoma, Morvan syndrome	Myasthenia gravis	MG-ADL = 8	N/T	N/T	N/T	N/T	N/T
SRPA01	26	Female	hyperthyroidism	Wilson disease	GCS = 9	53	64	32	176	0.9

ASAT – Aspartate aminotransferase, ALAT – Alanine aminotransferase, CRP – C-reactive protein, GBS-DS – Guillain-Barré Syndrome disability score [34], GCS – Glasgow coma scale [36], LDH – Lactate dehydrogenase, MG-ADL – Myasthenia gravis activities of daily living scale [35], N/T – not tested, WD – Wilson disease.
